# Extensive culturomics of 8 healthy samples enhances metagenomics efficiency

**DOI:** 10.1371/journal.pone.0223543

**Published:** 2019-10-21

**Authors:** Ami Diakite, Grégory Dubourg, Niokhor Dione, Pamela Afouda, Sara Bellali, Issa Isaac Ngom, Camille Valles, Matthieu Million, Anthony Levasseur, Frédéric Cadoret, Jean-Christophe Lagier, Didier Raoult

**Affiliations:** 1 Aix Marseille University, IRD, AP-HM, MEPHI, Marseille, France; 2 IHU Méditerranée Infection, Marseille, France; 3 Assistance Publique-Hôpitaux de Marseille, Marseille, France; South Dakota State University, UNITED STATES

## Abstract

Molecular approaches have long led to the assumption that the human gut microbiota is dominated by uncultivable bacteria. The recent advent of large-scale culturing methods, and in particular that of culturomics have demonstrated that these prokaryotes can in fact be cultured. This is increasing in a dramatic manner the repertoire of commensal microbes inhabiting the human gut. Following eight years of culturomics approach applied on more than 900 samples, we propose herein a remake of the pioneering study applying a dual approach including culturomics and metagenomics on a cohort of 8 healthy specimen. Here we show that culturomics enable a 20% higher richness when compared to molecular approaches by culturing 1 archaeal species and 494 bacterial species of which 19 were new taxa. Species discovered as a part of previous culturomics studies represent 30% of the cultivated isolates, while sequences derived from these new taxa enabled to increase by 22% the bacterial richness retrieved by metagenomics. Overall, 67% of the total reads generated were covered by cultured isolates, significantly reducing the hidden content of sequencing methods compared to the pioneering study. By redefining culture conditions to recover microbes previously considered fastidious, there are greater opportunities than ever to eradicate metagenomics dark matter.

## Introduction

The intestinal microbiota corresponds to a set of microorganisms that colonize the digestive tract. Its importance for human health is currently under the spotlight, as several studies have already reported its crucial role in the maintenance of human health but also in disease [1,2], as yielded by the fecal microbiota transplantation (FMT) during *Clostridium difficile* infections [3]. The first studies investigating the exploration of gut microbiota based on molecular approaches highlighted the diversity of the human gut microbiota but also that a large part (i.e. 80%) of the bacterium was “uncultivable” [4]. In order to culture the “uncultivable”, the culturomics approach was initiated and has proven over the last 5 years that these unknown species are rather “uncultured yet” [5]. The first culturomics study based on 212 culture conditions in 2012 by Lagier et al. has allowed to add 174 bacterial species to the repertoire of the Human Gut, including 31 new species and new bacterial genera [6]. In 2014, Dubourg et al. selected 70 culture conditions that covered the species identified by the previous 212 and continued to enrich the repertoire of the human gut by identifying unknown bacterial species from the gut [7]. Lagier et al. have highlighted the contribution of culturomics to the exploration of the human gut in 2016. Indeed, based on the selection of the 18 best culture conditions in order to explore a larger number of samples, this study considerably broadened the repertoire of the human gut by adding 531 bacterial species, including 197 new species and bacterial genera [8]. This technique was found to be complementary to metagenomics [5,8] to capture the prokaryotic diversity from human gut. As a matter of fact, between 2015 and 2018 culturomics has contributed up to 66.2% towards updating the repertoire of cultured bacteria from human being [9]. In addition, it partially fills gaps in metagenomics since the genomes of some new species isolated by culturomics actually correspond to certain sequences identified by metagenomics and defined as unassigned and non-cultivable in previous studies [8]. The addition of these new species genomes to the metagenomics database improves the potential of metagenomics to identify bacterial species. These results are the fruit of the culturomics approach applied to more than 900 samples. Hence, we propose as a remake of the pioneering study [6] to assess how culturomics studies have contributed to reducing the unknown share of metagenomics studies by using a dual approach (i.e. using culture-dependent and independent techniques) applied to a cohort of 8 apparently healthy subjects.

## Materials and methods

### Sample collection

A total of 8 stool samples were included as a part of this study. The specimens were collected from apparently healthy subjects, including several donors included as a part of fecal microbiota transplantation (FMT) [10,11] The subjects have been living in Marseille for at least a year but may come from different countries.

The fresh stool samples were directly inoculated 5 minutes after emission to prevent the loss of certain anaerobic bacteria by preservation. Samples were collected at different times to test the maximum culture condition. The donors have all signed a written consent and the project has been approved by the IHU Méditerranée Infection's ethics committee under number 2016–011. Individuals did not take antibiotics or other treatment at the time of collection. The main informations are summarized in Table 1.

**Table 1:** Characteristics from the donors sampled as a part of this study.

Donors	Size (m)	Weight (Kg)	Body mass index	Age (years)	Gender	Origin
Megagut 1	1,65	60	22	28	Female	French
Megagut 2	1,8	72	22,2	32	Male	Senegalese
Megagut 3	1,84	76	22,4	32	Male	Senegalese
Megagut 4	1,75	55	18	27	Female	Beninese
Megagut 5	1,79	95	29,6	32	Male	Cameroon
Megagut 6	1,7	69	23,6	30	Male	French
Megagut 7	1,65	70	25,7	27	Female	Algerian
Megagut 8	1,78	82	25,9	27	Male	Algerian

### Culturomics approach

Culturomics is a high-throughput culture technique consisting in the multiplication of the culture conditions along with a quick bacterial identification using MALDI-TOF MS [6]. MALDI-TOF has demonstrated its efficiency in bacterial identification in both clinical microbiology labs and culturomics studies [8,12]. Herein, a total of 58 culture conditions were tested (S1 Table). Of these, we have included the the18 best culturomics conditions previously selected [8], 18 new media and conditions of culture, some of which have been developed to grow certain anaerobic, fastidious and slow-growing bacteria and 22 alcohol conditions, whose purpose is to selectively isolate spore-forming bacteria according to the protocol of Browne et al. [13]. After emission, 1g of each sample is diluted in a 900 μl solution of Dulbecco's Phosphate-Bufered Saline (DPBS) and then immediately inoculated into a culture flask as previously described. Bacterial cultures are monitored for 30 days. Every 3 days, the liquids of blood culture flasks are inoculated with Colombia agar enriched with sheep blood at 5% (bioMérieux, Marcy l'Etoile, France) after series of dilutions. For direct inoculations without pre-enrichment, the samples diluted in phosphate buffered saline (PBS) undergo a series of dilutions before being seeded on the different solid media.

Archaeal species culture, in particular *Methanobrevibacter smithii* was performed by a co-culture technique as described by Traore et al. [14].

### Bacterial identification by MALDI-TOF/MS and 16s Ribosomal RNA sequencing

After 24 to 72 hours of incubation, the bacterial colonies obtained are identified by MALDI-TOF according to the protocol described by Seng et al. [12]. Bacterial colonies unidentified by MALDI-TOF mass spectrometry were sequenced with 16S ribosomal RNA, as previously described [15]. Following 16S sequencing, sequences that have a similarity percentage lower than 98.65% are defined as new bacterial species and those less than 95% as new bacterial genera [16]. These are described according to the taxonogenomic principle described by Fournier et al. [17].

### Metagenomics

#### Sequencing

The DNA from the 8 Megagut samples was extracted using two protocol (i.e., using a protease step and a deglycosylation step respectively) and further amplified pooled and barcoded, then sequenced for 16S rRNA sequencing on MiSeq technology (Illumina, Inc, San Diego CA 92121, USA). For each protocol extraction, metagenomic DNA was amplified for the 16S “V3-V4” regions by PCR for 40 cycles, using the Kapa HiFi Hotstart ReadyMix 2x (Kapa Biosystems Inc,Wilmington, MA U.S.A), and the surrounding conserved region V3_V4 primers with overhang adapters:

(FwOvAd_341F TCGTCGGCAGCGTCAGATGTGTATAAGAGACAGCCTACGGGNGGCWGCAG; RevOvAd_785R GTCTCGTGGGCTCGGAGATGTGTATAAGAGACAGGACTACHVGGGTATCTAATCC

After purification on AMPure beads (Beckman Coulter Inc, Fullerton, CA, USA), concentration was measured using High sensitivity Qubit technology (Beckman Coulter Inc, Fullerton, CA, USA) and dilution to 1 ng/μl was performed. At this step, library of protocol 1 was pooled volume to volume to library for protocol 5: so that 15ng were involved in a subsequent limited cycle PCR, where Illumina sequencing adapters and dual-index barcodes were added to the amplicon. After purification on AMPure beads (Beckman Coulter Inc, Fullerton, CA, USA), this library was pooled with 95 other multiplexed samples. The global concentration was quantified by a Qubit assay with the high sensitivity kit (Life technologies, Carlsbad, CA, USA). Before loading for sequencing on MiSeq (Illumina Inc, San Diego, CA, USA) the pool was diluted at 7pM. The 8 samples were also sequenced with paired end strategy according to the Nextera XT DNA sample prep kit (Illumina). To prepare the library, the « tagmentation » step fragmented and tagged 1ng of input DNA. Then, limited cycle PCR amplification (12 cycles) completed the tag adapters and introduced dual-index barcodes. After purification on AMPure XP beads (Beckman Coulter Inc, Fullerton, CA, USA), the libraries were normalized on specific beads according to the Nextera XT protocol (Illumina) then pooled for sequencing on the MiSeq. For both strategies, automated cluster generation and paired end sequencing with dual index reads were performed in a single 39-hours run in 2x250-bp.

#### Data processing: filtering the reads, dereplication and clustering

The paired-end reads of the corresponding raw fastq files were assembled into contigs using Pandaseq [18]. Then high-quality sequences were selected by considering only sequences that contained both primers (forward and reverse). Sequences containing N or those with a length shorter than 100 nt were then removed, and sequences longer than 500 nt were trimmed. Both forward and reverse primers were also removed from each of the sequences. The filtering steps were performed using the QIIME pipeline [19]. Strict dereplication (clustering of duplicate sequences) was performed on the filtered sequences, and they were then sorted by decreasing number of abundance [20–22]. For each metagenome, the clustering of OTUs was performed with 97% identity.

#### Building reference databases

We downloaded the Silva SSU and LSU database and release 1.32 from the Silva website and, from this, a local database of predicted amplicon sequences was built by extracting the sequences containing both primers. We have also added to this database the 16S sequences from 556 species isolated in our laboratory isolated as a part of the diagnosis or by culturomics. We obtained a reference database of 14,459 sequences. We also added all the putative species from our previous analyzes resulting a final database containing 76,368 sequences to perform our analysis.

#### Taxonomic assignments

We have applied at least 20 reads per OTU for the taxonomic assignment. The OTUs were then searched against each database using BLASTN [23]. The best match of ≥ 97% identity and 100% coverage for each of the OTUs was extracted from the reference database, and taxonomy was assigned up to the species level. Finally, we counted the number of OTUs assigned to unique species.

## Results

### Sample characteristics

The mean age of the 8 donors from different countries was of 29, with a mean body mass index of 24. The sex ratio was 1.6 in favor of the male gender (Table 1).

### Culturomics

**Fig 1:**
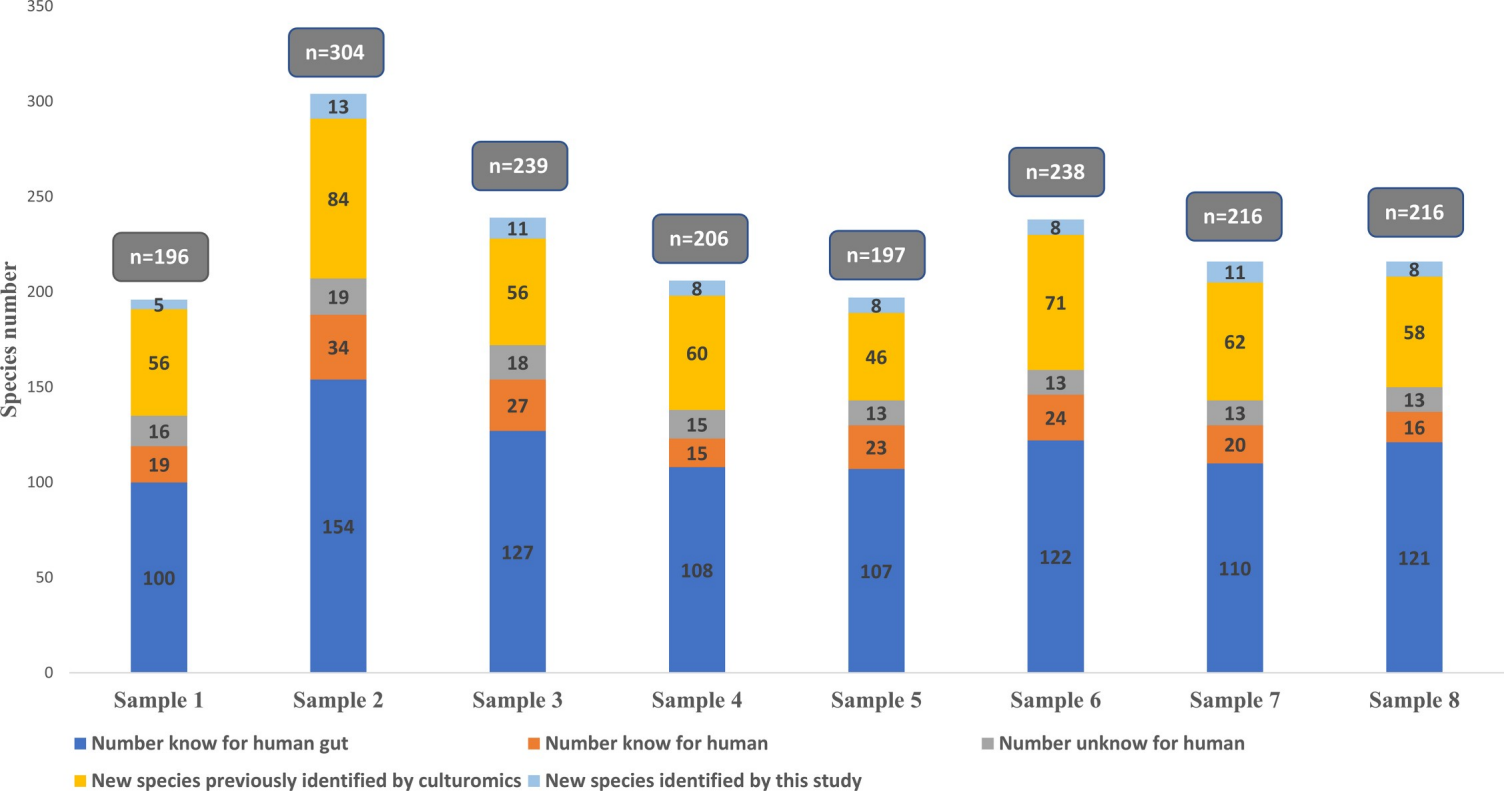
Number of isolated species by sample in culturomics.

The 58 culture conditions (S1 Table) used for the analysis of the 8 samples in this study allowed a total of 238,092 colonies to be analyzed by MALDI-TOF MS with an average of 29,762 colonies per sample. This resulted in the identification of 494 different bacterial and 1 Archaeal species (*Methanobrevibacter smithii*). Of these, 240 (48%) were known to the human gut, 56 (11%) were known to human but not in the gut, 35 (7%) were unknown to humans, and 146 (30%) were species previously discovered as a part of culturomics studies. In addition, 19 (4%) were isolated for the first time as part of the present study. Regarding their tolerance to oxygen, 68% of the bacterial species isolated in this work are strictly anaerobic and 32% are tolerant to oxygen (Fig 1, S2 Table).

Bacteria were classified into eight phyla. The most common phyla in these samples were *Firmicutes* (64%, n = 316), followed by *Bacteroidetes* (15%, n = 74), *Actinobacteria* (14%, n = 69) and *Proteobacteria* (6%, n = 31). We also isolated the bacterial species from rare phyla, such as *Synergistetes* (2 species), *Fusobacteria* (2 species), *Verrucomicrobia* (1 species) and *Lentisphaerae* (1 species) (S1 Fig). Bacterial species found are divided into 184 genera for which that of *Clostridium (*8%, n = 40), *Lactobacillus* (6%, n = 29), *Bacteroides* (5%, n = 23), *Streptococcus* (3%, n = 17) and *Bacillus* (3%, n = 16) are the five most represented (S1 Fig).

### Metagenomics

**Fig 2:**
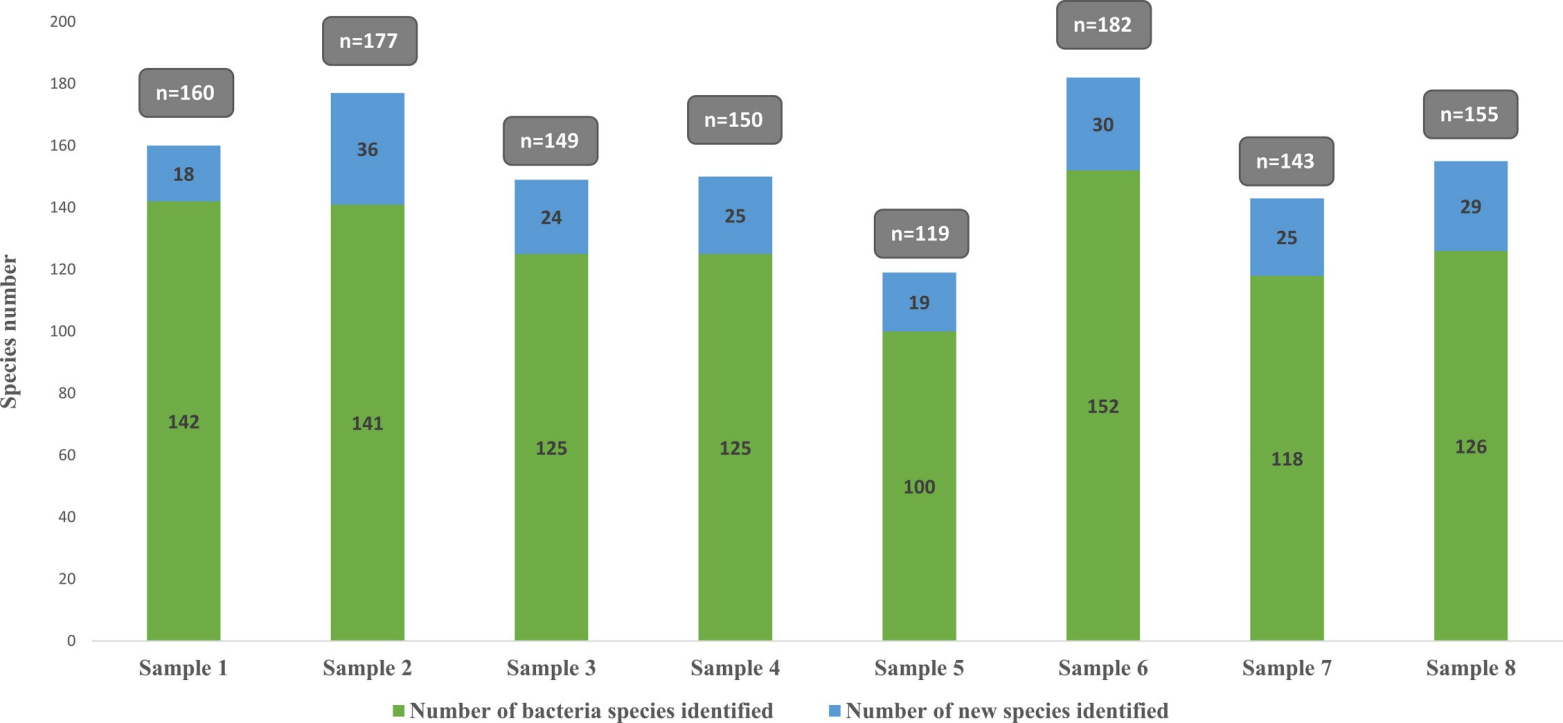
Number of identified species by metagenomics and distribution of associated reads percentage. Grey bar represents the percentage of bacterial species identified as species level, including those first isolated by culturomics in dark grey.

The analysis of 8 samples by the metagenomics generated a total of 826,080 reads, with an average of 103,260 reads per sample. Of these, 535,041 reads (65%) were associated with known species and allowed the identification of 362 different identified species including 2 archaeal species and 81 (22%) species previously discovered as a part of culturomics studies. The species previously discovered as a part of culturomics studies generated 95,652 reads which represent 18% of the total reads identified as species level. When considering the OTUs identified at the genus level, 68% of bacteria are strictly anaerobic and 32% are oxygen-tolerant (Fig 2, S3 Table).

Seven bacterial phyla are found by metagenomics: *Firmicutes* (66%, n = 1185), followed by *Bacteroidetes* (15%, n = 266), *Actinobacteria* (11%, n = 205), *Proteobacteria* (7%, n = 119), *Verrucomicrobia* (1%, n = 9), *Fusobacteria* (0%, n = 5) and *Elusimicrobia* (0%, n = 2) (S2 Fig). Bacterial species found are divided into 196 genera with *Bacteroides* (10%, n = 105), *Clostridium* (6%, n = 61), *Ruminococcus* (5%, n = 56), *Eubacterium* (5%, n = 53), and *Blautia (*4%, n = 40) in top 5 (S2 Fig). In addition, 2 Archeal species (i.e., *Methanobrevibacter smithii* and *Methanosphaera stadtmanae*) belonging to *Euryarchaeota* phyla have been identified.

### Comparison between culturomics and metagenomics

**Fig 3:**
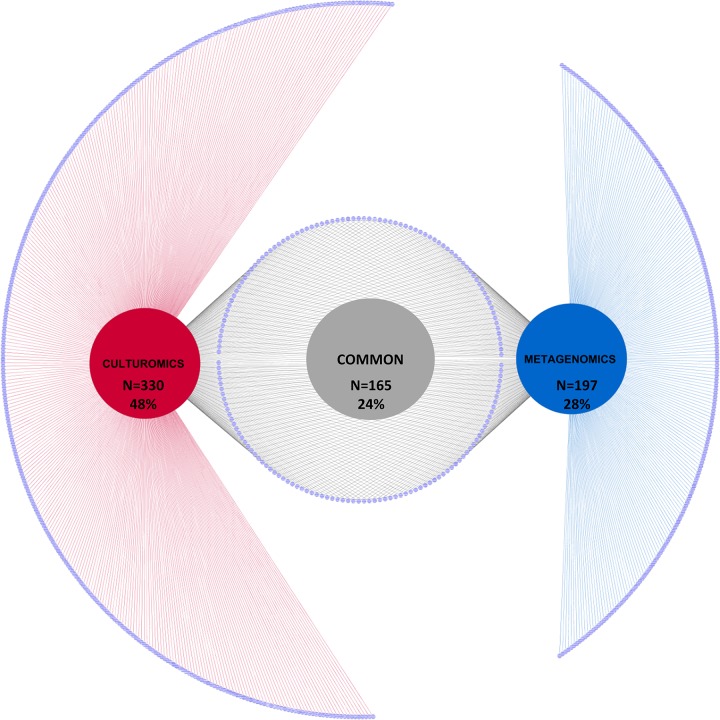
Comparison of bacterial species identified by metagenomics vs culturomics. The small violet circle represents bacteria species.

A total of 690 bacterial species and 2 archaeal species were identified in this study using the culture-independent or culture-dependant approach. In detail, culturomics allowed the isolation of 494 bacterial species and 1 archaeal species, while metagenomics detected 360 bacterial species and 2 archaeal species. The overlap between the two approaches at the species level was of 24%, corresponding to 165 species. (Fig 3).

However, the number of species identified only by culturomics (48%, n = 330) was 20% higher than that identified by metagenomics (28%, n = 197).

**Fig 4:**
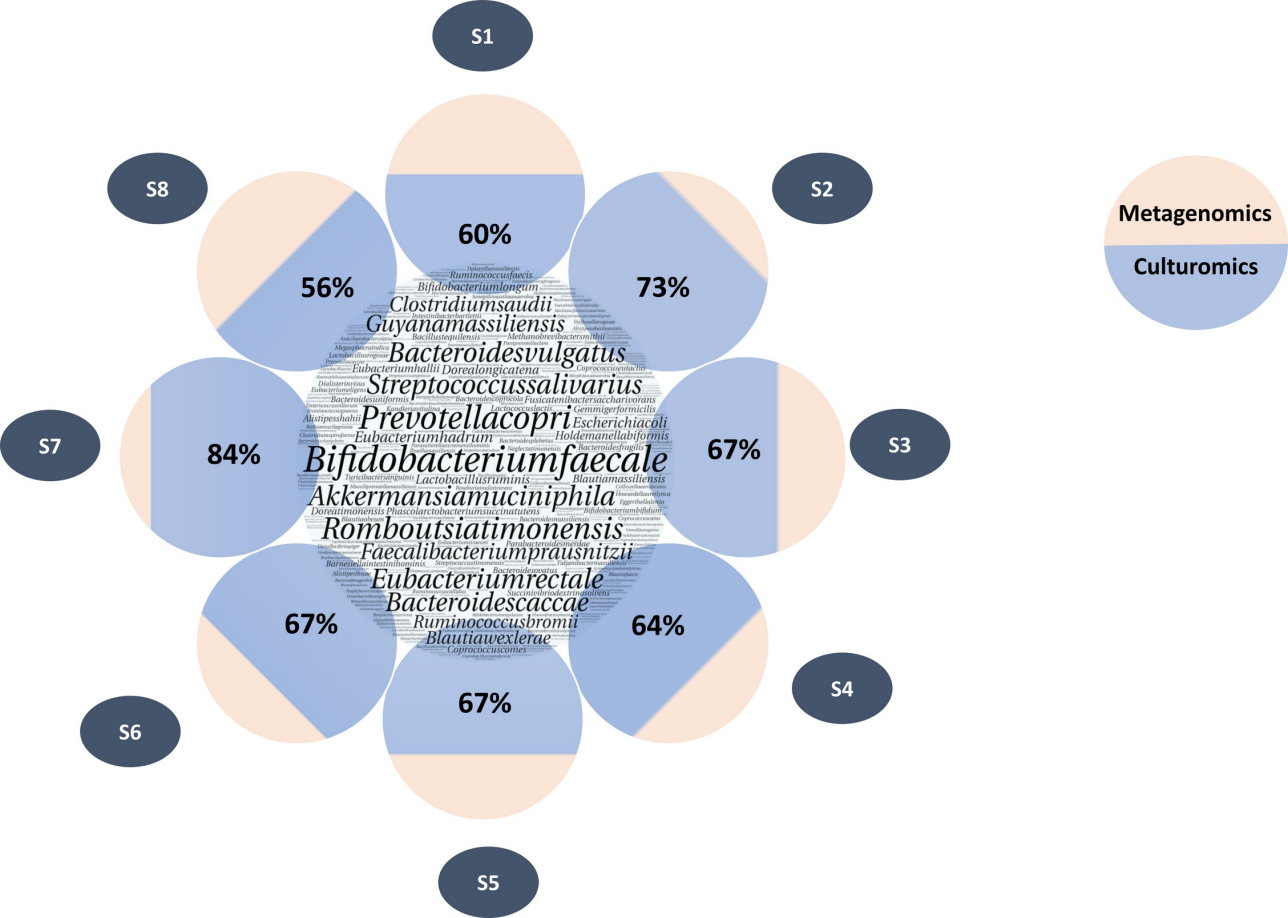
Comparison of read percentages of species identified by culturomics vs metagenomics. The large circle in the center represents all species identified by metagenomics. The circles around them represent each sample. Blue part represents the percentage of reads corresponding to the species identified by culturomics. In beige, the remaining percentage of reads assigned to species level by metagenomics.

When comparing these two approaches regarding the proportion of reads detected, species recovered by culturomics represented 365,079 reads for the 167 species common to both techniques. This represents 68% of the total number of reads identified at the species level by metagenomics. When looking in detail for each sample, the average proportion of the total number of readings per sample detected by metagenomics and also recovered by culturomics was 67% (Figs 3 and 4).

### Core microbiome

We aimed to identify the bacterial taxa present among all individuals. Culturomics allowed to isolate 45 bacterial species (9%) common to all the samples, shared in 4 phyla: *Firmicutes* (49%), *Bacteroidetes* (29%), *Actinobacteria* (20%), *Proteobacteria* (2%) and 23 genera for which *Clostridium (*20%), *Alistipes* (13%), *Bacteroides* (9%), *Bifidobacterium* (7%), *Enterococcus* (7%) and *Lactobacillus* (7%) are the most common. Of the 45 species in common, 80% are strictly anaerobic and 18% (n = 8) were species previously discovered by culturomics.

Regarding metagenomics, 46 different OTU assigned at the species level (13%) are common to all samples, shared in 5 Phylum: *Firmicutes* (72%), *Bacteroidetes* (13%), *Actinobacteria* (11%), *Proteobacteria* (2%), *Fusobacteria* (2%) and 28 genera: *Blautia* (11%), *Bacteroides* (7%), *Coprococcus* (7%), *Eubacterium* (7%), *Ruminococcus* (7%) are the most common. Of the 46 bacterial species detected in all samples, 72% are strictly anaerobic and 22% (n = 10) represented species previously discovered by culturomics.

Taken together, the two approaches identified a total of 88 species common to our samples, only 3 of which were found with both methods.

### Contribution of the culturomics approach of the present study in the human intestinal microbiota study

Of the 494 bacterial species recovered by culture, 165 (34%) were discovered for the first time in culturomics studies. Of these, 119 (77%) were found in at least 2 samples, of which 59 (50%) were found in half of the samples. In addition, 8 were common to all samples (*Alistipes jeddahensis*, *Alistipes obesi*, *Eubacterium massiliense*, *Holdemania massiliensis*, *Marseillibacter massiliensis*, *Oscilibacter massiliensis*, *Peptoniphilus grossensis and Raoultibacter massiliensis*).

Regarding metagenomics, of the 360 species identified, 81 (22%) were discovered for the first time as a part of the culturomics studies and represents 18% of the number of reads assigned to the species level. This highlights the role of culturomics in decreasing the size of the “dark matter”. Besides, 54 (67%) species were found in at least 2 samples, of which 30 (55%) were found in half of this samples. In addition, 10 new species classified by the abundance of their number of reads were present in all samples (*Romboutsia timonensis*, *Clostridium saudii*, *Blautia massiliensis*, *Dorea timonensis*, *Streptococcus timonensis*, *Neglecta timonensis*, *Lachnoclostridium bouchesdurhonense*, *Holdemania timonensis*, *Lachnoclostridium edouardi and Blautia mediterranea*). A total of 18 species, identified for the first time by culturomics, are common to all samples according to the metagenomics and culturomics approaches, of which no species are common to both methods. This shows that at least some of the new species found by culturomics are in fact common gut commensals.

By comparing the bacterial species isolated in this study with those of other previous studies on culturomics on gut, including those of Lagier et al. [8], Bilen et al. [9] and Mailhe et al. [24], this present work adds 113 species to the repertoire of the human gut identified by culturomics culture. A total of 1465 bacterial species have now been identified in the human gut by culturomics, including 435 (30%) species isolated for the first time by this approach.

## Discussion

The primary goal of this study was the exploration and description of the human gut microbiota of healthy individuals through metagenomics and culturomics. This work has led to the global identification of 690 bacterial species and 2 archaeal species when the two approaches are combined, the majority of which are anaerobic (60%).

Phylum and genera distribution are concordant with figures found in the literature[9,24].

Although the donors are of different genders and especially of different origins, a core microbiome of 88 bacterial species have been established when combining culturomics and metagenomics, 20% of which is composed of species previously discovered by culturomics. Considering that these new species have been isolated for the first time in samples from various geographical areas, they are still found among our 8 healthy donors of diverse origins. This finding suggest that these new species are not specific to a specific area and are in fact very common. This is comforted by the fact that one third of the species identified by culture in this study were previously discovered as part of culturomics studies. The second lesson drawn from these data is that culturomics studies have increased by 20% the number of species that could be identified by metagenomics, thus reflecting a circular complementary between the two approaches. Above and beyond, culturomics is able to capture at the species level a higher bacterial richness than metagenomics. If these results are in line with other publications [7,25] that focused on atypical specimen, we have however included in this study only healthy subjects. Indeed, this is the first time so far that culture has surpassed high throughput sequencing methods to extract bacterial diversity from complex communities. Also, regarding of the number of reads, the culturomics could challenge metagenomics approach, as 67% of the total number of reads in each sample correspond to species recovered by culturomics. In addition, culturomics still allows to go beyond the depth bias of sequencing methods proving that the method is extremely sensitive as i) 27 bacterial species with less than 5 reads have been isolated by culturomics, and ii) 329 species were only identified by culture.

## Conclusion

Finally, eight years after the first culturomics studies, time for assessment has come. Indeed, we have previously highlighted the dramatic expansion of the bacterial repertoire associated with the human gut, but also with other human sites. As part of this study, 19 new supplementary taxa were discovered, thereby pursuing the expansion of the microbial repertoire colonizing human being. Most importantly, a dramatic decrease of sequences attributed to the “uncultivable” was evidenced in this study. Indeed, 80% of the bacteria were considered as such before the pioneering culturomics study, while we demonstrate herein that culture-dependent approach allows to capture 67% of the diversity identified by metagenomics regarding the number of reads. This spectacular paradigm shift should encourage us to pursue our efforts to culture the unknown and challenge sequencing approaches. One of the possible explanation of the sequences derived from metagenomes for which no corresponding isolate was found by culture could be due to a viability issue. Thus, emerging tools are currently under development to discriminate live from dead bacteria among complex ecosystems [26]. As the human gut is constantly exposed to environmental bacteria in particular through food, this would allow a precise definition of what is a commensal and what is only passing through the gut. Finally, substantial improvements could be made to improve efficiency of culture methods. Throughout the culturomics studies, we mainly focalized on bacteria considered fastidious. However, several works support the hypothesis that the isolation of “fastidious” taxa could be influenced by very subtle changes in culture methods [27,28], and others showing that the isolation of rare microorganisms could be just the result of chance [29] or *in extenso* of high-scale screening. Taken together, these elements provide exciting perspectives aiming to complete the eradication of the metagenomics dark matter.

## Supporting information

S1 Table List of culture condition used in this study. (XLSX)

S2 Table List of bacteria identified by Culturomics. (XLSX)

S3 Table List of bacteria identified at the species level by metagenomics. (XLSX)

S1 Fig A: Distribution of bacterial species into 8 main phylum in culturomics. B: Distribution of bacterial species into 184 genera in culturomics. This figure is generated thanks to the WordArt online tool (wordart.com). The size of the denomination of each genus is proportional to its frequency in this study.
(PDF)

S2 Fig A: Distribution of bacterial species into 7 main phylum in metagenomics. B: Distribution of bacterial species into 196 genera in metagenomics. This figure is generated thanks to the WordArt online tool (wordart.com). The size of the denomination of each genus is proportional to its frequency in this study.
(PDF)

## References

[1] Dhakan DB, Maji A, Sharma AK, Saxena R, Pulikkan J, Grace T, et al The unique composition of Indian gut microbiome, gene catalogue, and associated fecal metabolome deciphered using multi-omics approaches. GigaScience. 2019;8 10.1093/gigascience/giz004 30698687 PMC6394208

[2] Sekirov I, Russell SL, Antunes LCM, Finlay BB. Gut microbiota in health and disease. Physiol Rev. 2010;90: 859–904. 10.1152/physrev.00045.2009 20664075

[3] Rao K, Young VB. Fecal Microbiota Transplantation for the Management of Clostridium difficile Infection. Infect Dis Clin North Am. 2015;29: 109–122. 10.1016/j.idc.2014.11.009 25677705 PMC4328137

[4] Eckburg PB, Bik EM, Bernstein CN, Purdom E, Dethlefsen L, Sargent M, et al Diversity of the Human Intestinal Microbial Flora. Science. 2005;308: 1635–1638. 10.1126/science.1110591 15831718 PMC1395357

[5] Lagier J-C, Hugon P, Khelaifia S, Fournier P-E, La Scola B, Raoult D. The Rebirth of Culture in Microbiology through the Example of Culturomics To Study Human Gut Microbiota. Clin Microbiol Rev. 2015;28: 237–264. 10.1128/CMR.00014-14 25567229 PMC4284300

[6] Lagier J-C, Armougom F, Million M, Hugon P, Pagnier I, Robert C, et al Microbial culturomics: paradigm shift in the human gut microbiome study. Clin Microbiol Infect Off Publ Eur Soc Clin Microbiol Infect Dis. 2012;18: 1185–1193. 10.1111/1469-0691.12023 23033984

[7] Dubourg G, Lagier JC, Robert C, Armougom F, Hugon P, Metidji S, et al Culturomics and pyrosequencing evidence of the reduction in gut microbiota diversity in patients with broad-spectrum antibiotics. Int J Antimicrob Agents. 2014;44: 117–124. 10.1016/j.ijantimicag.2014.04.020 25063078

[8] Lagier J-C, Khelaifia S, Alou MT, Ndongo S, Dione N, Hugon P, et al Culture of previously uncultured members of the human gut microbiota by culturomics. Nat Microbiol. 2016;1: 16203 10.1038/nmicrobiol.2016.203 27819657 PMC12094094

[9] Bilen M, Dufour J-C, Lagier J-C, Cadoret F, Daoud Z, Dubourg G, et al The contribution of culturomics to the repertoire of isolated human bacterial and archaeal species. Microbiome. 2018;6: 94 10.1186/s40168-018-0485-5 29793532 PMC5966928

[10] Hocquart M, Lagier J-C, Cassir N, Saidani N, Eldin C, Kerbaj J, et al Early Fecal Microbiota Transplantation Improves Survival in Severe Clostridium difficile Infections. Clin Infect Dis Off Publ Infect Dis Soc Am. 2018;66: 645–650. 10.1093/cid/cix762 29020328

[11] Saïdani N, Lagier J-C, Cassir N, Million M, Baron S, Dubourg G, et al Fecal microbiota transplantation shortens the colonization period and allows the re-entry of patients carrying carbapenamase-producing bacteria into medical care facilities. Int J Antimicrob Agents. 2018; 10.1016/j.ijantimicag.2018.11.014 30472293

[12] Seng P, Drancourt M, Gouriet F, La Scola B, Fournier P-E, Rolain JM, et al Ongoing revolution in bacteriology: routine identification of bacteria by matrix-assisted laser desorption ionization time-of-flight mass spectrometry. Clin Infect Dis Off Publ Infect Dis Soc Am. 2009;49: 543–551. 10.1086/600885 19583519

[13] Browne HP, Forster SC, Anonye BO, Kumar N, Neville BA, Stares MD, et al Culturing of “unculturable” human microbiota reveals novel taxa and extensive sporulation. Nature. 2016;533: 543–546. 10.1038/nature17645 27144353 PMC4890681

[14] Traore SI, Khelaifia S, Armstrong N, Lagier JC, Raoult D. Isolation and culture of Methanobrevibacter smithii by co-culture with hydrogen-producing bacteria on agar plates. Clin Microbiol Infect Off Publ Eur Soc Clin Microbiol Infect Dis. 2019; 10.1016/j.cmi.2019.04.008 30986553

[15] Morel A-S, Dubourg G, Prudent E, Edouard S, Gouriet F, Casalta J-P, et al Complementarity between targeted real-time specific PCR and conventional broad-range 16S rDNA PCR in the syndrome-driven diagnosis of infectious diseases. Eur J Clin Microbiol Infect Dis Off Publ Eur Soc Clin Microbiol. 2015;34: 561–570. 10.1007/s10096-014-2263-z 25348607

[16] Kim M, Oh H-S, Park S-C, Chun J. Towards a taxonomic coherence between average nucleotide identity and 16S rRNA gene sequence similarity for species demarcation of prokaryotes. Int J Syst Evol Microbiol. 2014;64: 346–351. 10.1099/ijs.0.059774-0 24505072

[17] Fournier P-E, Lagier J-C, Dubourg G, Raoult D. From culturomics to taxonomogenomics: A need to change the taxonomy of prokaryotes in clinical microbiology. Anaerobe. 2015;36: 73–78. 10.1016/j.anaerobe.2015.10.011 26514403

[18] Masella AP, Bartram AK, Truszkowski JM, Brown DG, Neufeld JD. PANDAseq: paired-end assembler for illumina sequences. BMC Bioinformatics. 2012;13: 31 10.1186/1471-2105-13-31 22333067 PMC3471323

[19] Caporaso JG, Kuczynski J, Stombaugh J, Bittinger K, Bushman FD, Costello EK, et al QIIME allows analysis of high-throughput community sequencing data. Nat Methods. 2010;7: 335–336. 10.1038/nmeth.f.303 20383131 PMC3156573

[20] Stoeck T, Behnke A, Christen R, Amaral-Zettler L, Rodriguez-Mora MJ, Chistoserdov A, et al Massively parallel tag sequencing reveals the complexity of anaerobic marine protistan communities. BMC Biol. 2009;7: 72 10.1186/1741-7007-7-72 19886985 PMC2777867

[21] Mondani L, Piette L, Christen R, Bachar D, Berthomieu C, Chapon V. Microbacterium lemovicicum sp. nov., a bacterium isolated from a natural uranium-rich soil. Int J Syst Evol Microbiol. 2013;63: 2600–2606. 10.1099/ijs.0.048454-0 23264499

[22] Boissière A, Tchioffo MT, Bachar D, Abate L, Marie A, Nsango SE, et al Midgut microbiota of the malaria mosquito vector Anopheles gambiae and interactions with Plasmodium falciparum infection. PLoS Pathog. 2012;8: e1002742 10.1371/journal.ppat.1002742 22693451 PMC3364955

[23] Altschul SF, Gish W, Miller W, Myers EW, Lipman DJ. Basic local alignment search tool. J Mol Biol. 1990;215: 403–410. 10.1016/S0022-2836(05)80360-2 2231712

[24] Mailhe M, Ricaboni D, Vitton V, Gonzalez J-M, Bachar D, Dubourg G, et al Repertoire of the gut microbiota from stomach to colon using culturomics and next-generation sequencing. BMC Microbiol. 2018;18: 157 10.1186/s12866-018-1304-7 30355340 PMC6201554

[25] Dubourg G, Lagier JC, Armougom F, Robert C, Hamad I, Brouqui P, et al The proof of concept that culturomics can be superior to metagenomics to study atypical stool samples. Eur J Clin Microbiol Infect Dis Off Publ Eur Soc Clin Microbiol. 2013;32: 1099 10.1007/s10096-013-1843-7 23430196

[26] Emerson JB, Adams RI, Román CMB, Brooks B, Coil DA, Dahlhausen K, et al Schrödinger’s microbes: Tools for distinguishing the living from the dead in microbial ecosystems. Microbiome. 2017;5: 86 10.1186/s40168-017-0285-3 28810907 PMC5558654

[27] Tanaka T, Kawasaki K, Daimon S, Kitagawa W, Yamamoto K, Tamaki H, et al A hidden pitfall in the preparation of agar media undermines microorganism cultivability. Appl Environ Microbiol. 2014;80: 7659–7666. doi: 10.1128/AEM.02741-14 25281372 PMC4249246

[28] Kato S, Yamagishi A, Daimon S, Kawasaki K, Tamaki H, Kitagawa W, et al Isolation of Previously Uncultured Slow-Growing Bacteria by Using a Simple Modification in the Preparation of Agar Media. Appl Environ Microbiol. 2018;84 10.1128/AEM.00807-18 30030229 PMC6146985

[29] Kurm V, van der Putten WH, Hol WHG. Cultivation-success of rare soil bacteria is not influenced by incubation time and growth medium. PloS One. 2019;14: e0210073 10.1371/journal.pone.0210073 30629606 PMC6328151

