# Supplementary material for: Extensive culturomics of 8 healthy samples enhances metagenomics efficiency
Source: PLoS One. 2019 Oct 21;14(10):e0223543. doi: 10.1371/journal.pone.0223543 (PMC6802823; doi:10.1371/journal.pone.0223543)
Supplement: S1 Fig — A: Distribution of bacterial species into 8 main phylum in culturomics. B: Distribution of bacterial species into 184 genera in culturomics. This figure is generated thanks to the WordArt online tool (wordart.com). The size of the denomination of each genus is proportional to its frequency in this study. (PDF) [file pone.0223543.s004.pdf]

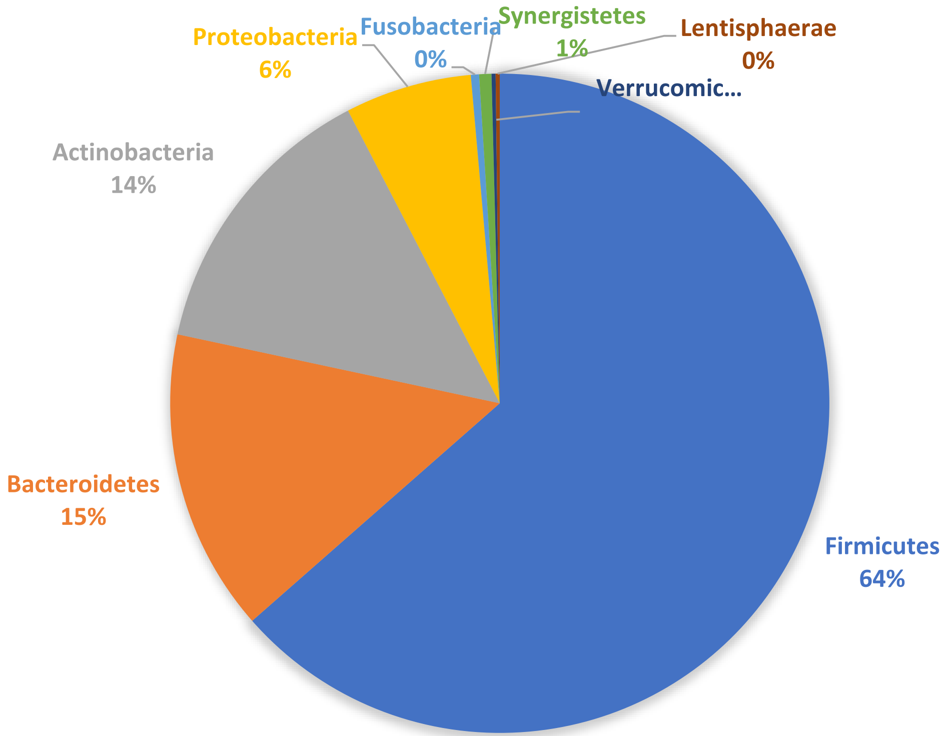

A. Distribution of bacterial species into 8 main phylum in culturomics

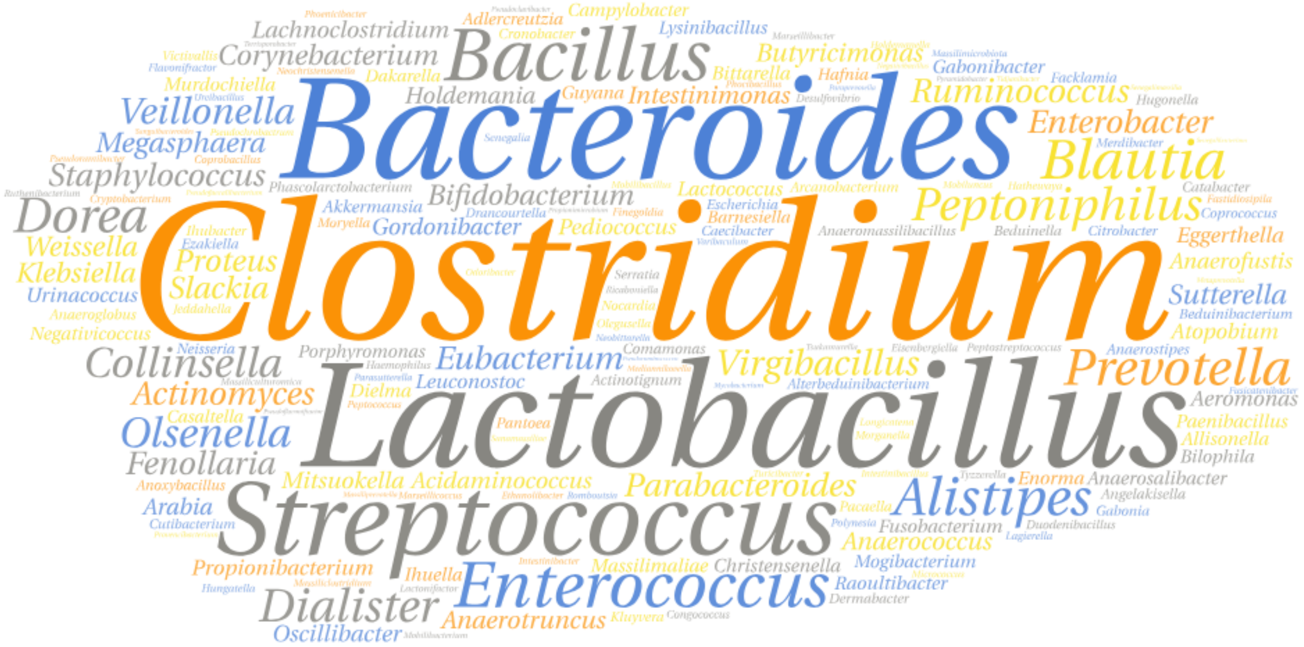

B. Distribution of bacterial species into 184 genera in culturomics. This figure is generated thanks to the WordArt online tool (wordart.com). The size of the denomination of each genus is proportional to its frequency in this study.
