# Supplementary material for: Extensive culturomics of 8 healthy samples enhances metagenomics efficiency
Source: PLoS One. 2019 Oct 21;14(10):e0223543. doi: 10.1371/journal.pone.0223543 (PMC6802823; doi:10.1371/journal.pone.0223543)
Supplement: S2 Fig — A: Distribution of bacterial species into 7 main phylum in metagenomics. B: Distribution of bacterial species into 196 genera in metagenomics. This figure is generated thanks to the WordArt online tool (wordart.com). The size of the denomination of each genus is proportional to its frequency in this study. (PDF) [file pone.0223543.s005.pdf]

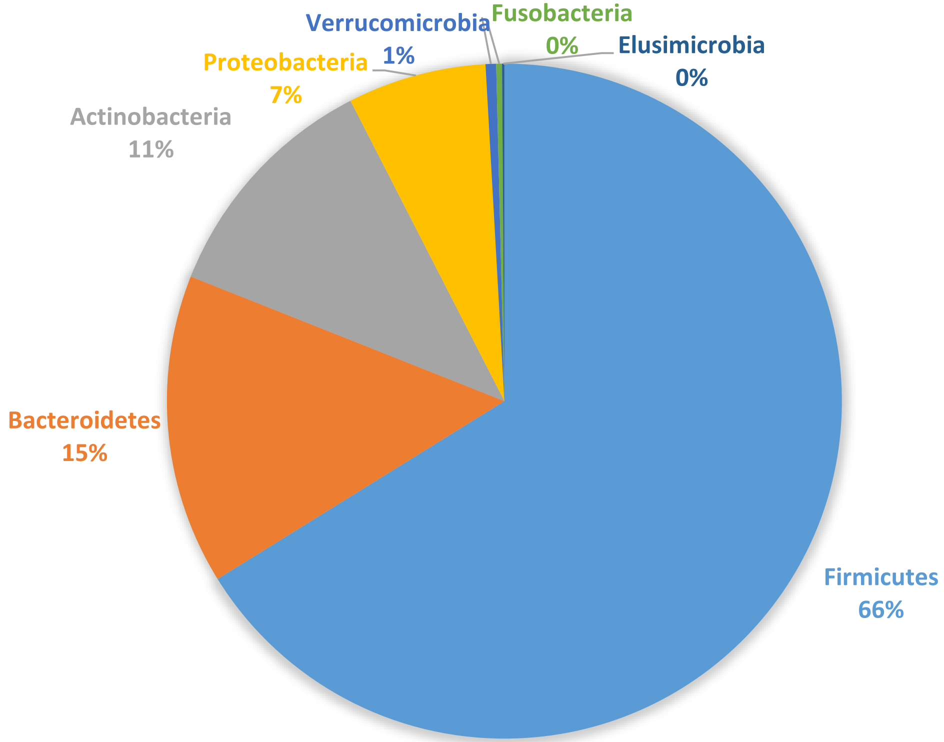

A. Distribution of bacterial species into 7 main phylum in metagenomics

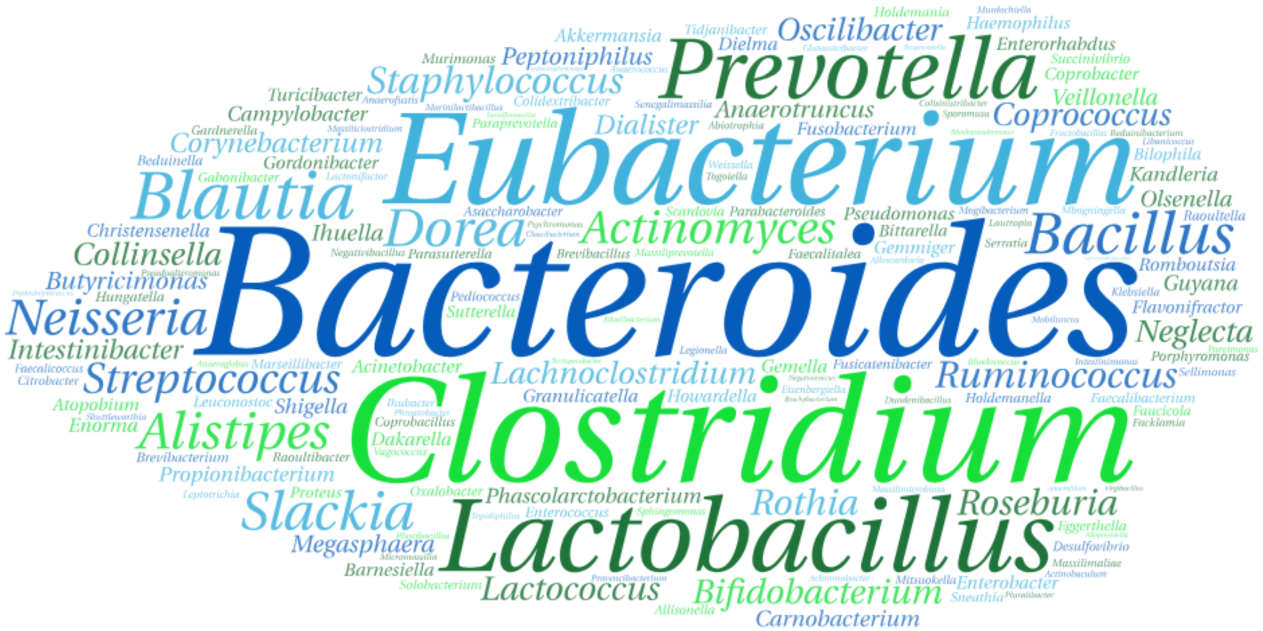

B. Distribution of bacterial species into 196 genera in metagenomics. This figure is generated thanks to the WordArt online tool (wordart.com). The size of the denomination of each genus is proportional to its frequency in this study.
